# Effect of Daytime versus Nighttime on Prehospital Care and Outcomes after Severe Traumatic Brain Injury

**DOI:** 10.3390/jcm13082249

**Published:** 2024-04-12

**Authors:** Carolien S. E. Bulte, Floor J. Mansvelder, Stephan A. Loer, Frank W. Bloemers, Dennis Den Hartog, Esther M. M. Van Lieshout, Nico Hoogerwerf, Joukje van der Naalt, Anthony R. Absalom, Saskia M. Peerdeman, Georgios F. Giannakopoulos, Lothar A. Schwarte, Patrick Schober, Sebastiaan M. Bossers

**Affiliations:** 1 Department of Anesthesiology, Amsterdam University Medical Center, Vrije Universiteit Amsterdam, 1081 HV Amsterdam, The Netherlands; f.j.mansvelder@amsterdamumc.nl (F.J.M.); s.loer@amsterdamumc.nl (S.A.L.); l.schwarte@amsterdamumc.nl (L.A.S.); p.schober@amsterdamumc.nl (P.S.); s.bossers@amsterdamumc.nl (S.M.B.); 2 Helicopter Emergency Medical Service Lifeliner 1, 1045 AR Amsterdam, The Netherlands; g.giannakopoulos@amsterdamumc.nl; 3 Department of Surgery, Amsterdam University Medical Center, Vrije Universiteit Amsterdam, 1081 HV Amsterdam, The Netherlands; fw.bloemers@amsterdamumc.nl; 4 Trauma Research Unit Department of Surgery, Erasmus MC, University Medical Center Rotterdam, 3015 GD Rotterdam, The Netherlands; d.denhartog@erasmusmc.nl (D.D.H.); e.vanlieshout@erasmusmc.nl (E.M.M.V.L.); 5 Department of Anesthesiology, Radboud University Medical Center, 6525 GA Nijmegen, The Netherlands; nico.hoogerwerf@radboudumc.nl; 6 Helicopter Emergency Medical Service Lifeliner 3, 5408 SM Volkel, The Netherlands; 7 Department of Neurology, University Medical Center Groningen, University of Groningen, 9713 GZ Groningen, The Netherlands; j.van.der.naalt@umcg.nl; 8 Department of Anesthesiology, University Medical Center Groningen, University of Groningen, 9713 GZ Groningen, The Netherlands; a.r.absolom@umcg.nl; 9 Department of Neurosurgery, Amsterdam University Medical Center, Vrije Universiteit Amsterdam, 1081 HV Amsterdam, The Netherlands

**Keywords:** traumatic brain injury, prehospital, mortality, outcome, emergency medical services

## Abstract

**Background/Objectives:** Severe traumatic brain injury (TBI) is a frequent cause of morbidity and mortality worldwide. In the Netherlands, suspected TBI is a criterion for the dispatch of the physician-staffed helicopter emergency medical services (HEMS) which are operational 24 h per day. It is unknown if patient outcome is influenced by the time of day during which the incident occurs. Therefore, we investigated the association between the time of day of the prehospital treatment of severe TBI and 30-day mortality. **Methods:** A retrospective analysis of prospectively collected data from the BRAIN-PROTECT study was performed. Patients with severe TBI treated by one of the four Dutch helicopter emergency medical services were included and followed up to one year. The association between prehospital treatment during day- versus nighttime, according to the universal daylight period, and 30-day mortality was analyzed with multivariable logistic regression. A planned subgroup analysis was performed in patients with TBI with or without any other injury. **Results:** A total of 1794 patients were included in the analysis, of which 1142 (63.7%) were categorized as daytime and 652 (36.3%) as nighttime. Univariable analysis showed a lower 30-day mortality in patients with severe TBI treated during nighttime (OR 0.74, 95% CI 0.60–0.91, *p* = 0.004); this association was no longer present in the multivariable model (OR 0.82, 95% CI 0.59–1.16, *p* = 0.262). In a subgroup analysis, no association was found between mortality rates and the time of prehospital treatment in patients with combined injuries (TBI and any other injury). Patients with isolated TBI had a lower mortality rate when treated during nighttime than when treated during daytime (OR 0.51, 95% CI 0.34–0.76, *p* = 0.001). Within the whole cohort, daytime versus nighttime treatments were not associated with differences in functional outcome defined by the Glasgow Outcome Scale. **Conclusions:** In the overall study population, no difference was found in 30-day mortality between patients with severe TBI treated during day or night in the multivariable model. Patients with isolated severe TBI had lower mortality rates at 30 days when treated at nighttime.

## 1. Introduction

Traumatic brain injury (TBI) is a major contributor to morbidity and mortality in trauma victims and is a leading cause of death worldwide in patients under 45 years [1,2]. The treatment of TBI without unnecessary delay, to prevent secondary brain injury, is of the utmost importance [3]. Prehospital care provided to this category of patients focusses on the prevention of secondary brain injury and typically includes securing the airway by endotracheal intubation, mechanical ventilation, hemodynamic stabilization and the administration of hyperosmotic therapy [4].

In the Netherlands, suspected severe TBI is a primary dispatch criterion for the helicopter emergency medical services (HEMS). Dutch HEMS is designed to transport a nurse and emergency physician to the patient as quickly as possible [5]. Based on more strict flight regulatory limits during darkness regarding weather conditions and limited landing options, the HEMS’ benefit of speed might be limited during nighttime. Moreover, individual and team performance may be hindered by fatigue and sleep deprivation during nighttime, similar to the in-hospital setting [6]. In addition, suboptimal work conditions for the medical team (e.g., limited visibility in a dark environment) and specific prehospital operational factors such as longer response times (e.g., due to more extensive flight preparation at nighttime) pose additional challenges and may affect outcome [7]. Finally, in the receiving hospitals, medical specialists may be on call from home during nighttime as opposed to daytime shifts which might influence the time from injury to definitive (surgical) care.

Whether the time of day of the accident and subsequent prehospital treatment of patients with severe TBI is associated with functional outcome or mortality is unknown. Evidence from previous studies is limited and conflicting. The admission of patients with severe TBI during office hours versus any other time had no influence on outcomes in adults [8,9]. However, one of the few studies also including prehospital care showed that the nighttime presentation of road traffic injury patients, including TBI, was associated with decreased survival in the emergency department compared to daytime [10]. The trauma system, available resources and logistical challenges may differ between countries and could hamper comparisons between studies.

The BRAIN-PROTECT (BRAin INjury: Prehospital Registry of Outcome, Treatments and Epidemiology of Cerebral Trauma) study is a large prospective multicenter observational cohort study performed in the Netherlands [11]. In this retrospective analysis of the BRAIN-PROTECT database, the association between the time of day of prehospital care for patients with severe TBI and patient outcome was studied. We hypothesized that nighttime treatment is associated with increased 30-day mortality compared with daytime treatment.

## 2. Materials and Methods

The present study is a retrospective analysis of the BRAIN-PROTECT study, a multicenter, prospective observational study on the prehospital treatment of patients with suspected severe TBI in the Netherlands. The complete study protocol was published previously [11]. According to the Medical Ethics Committees of the Amsterdam UMC, location VUmc (2012/041) and Erasmus MC Rotterdam (MEC-2012-515), this study was not subject to the Dutch Medical Research Involving Human Subjects Act and patient consent was waived. This analysis is reported according to the STROBE reporting guidelines.

### 2.1. Patient Selection

From February 2012 until December 2017, all patients with suspected severe TBI (prehospital GCS ≤ 8 and a trauma mechanism or injuries suggestive of TBI) treated by one of the four Dutch helicopter emergency medical services (HEMS) were included in the BRAIN-PROTECT cohort. All patients with suspected TBI were purposefully included because prehospital treatment is based on the suspicion of TBI rather than on a definitive diagnosis. Patients who underwent traumatic cardiopulmonary resuscitation prior to hospital admission were excluded since this patient category has a high mortality irrespective of the treatment of TBI.

### 2.2. Outcome Measures

Patients were transported to one of the nine participating level-1 trauma centers, and in-hospital and outcome data up to one year were collected. The primary outcome of this analysis was (all-cause) 30-day mortality. Secondary outcomes included the Glasgow Outcome Scale (GOS) at discharge, hospital length of stay (LOS) and specific prehospital outcomes, namely on-scene time and first-pass intubation rate.

### 2.3. Day- and Nighttime

For this analysis, patients with suspected severe TBI were categorized in day- versus nighttime treatment based on the time of dispatch during or after the uniform daylight period (UDP) respectively. The uniform daylight period commences 15 min before sunrise and ends 15 min after sunset. Throughout the year, the UDP changes with the seasons, and therefore, the UDP is not a fixed time period. The start and end of the UDP is an important time point in the Dutch HEMS operation and is registered precisely by the pilots. Hence, the UDP was considered a reliable variable and chosen to define day- and nighttime in this analysis also because the logistical modus operandi of Dutch HEMS alters after dark. The Dutch HEMS system is a 24/7 operational ambulance service staffed by a nurse, emergency physician, ambulance driver and helicopter pilot, scheduled in 12.5 h shifts. The system is designed to transport the team to the patient as quickly as possible, either by helicopter or ambulance vehicle. The mode of transportation is determined for each individual dispatch and is based on logistic, technical and meteorological factors. Subsequently, during both day- and nighttime, the vast majority of patients are transported by the ambulance and HEMS crew to the hospital using a regular road ambulance vehicle. In the Netherlands, travel distances to the hospital are relatively short, and transport by road ambulance is usually faster and more efficient than by helicopter. During the night, the HEMS team uses the road ambulance vehicle more often to travel to the incident than during the day due to more strict flight regulations which may influence the travel time to the patient. Another consequence of stricter regulations during nighttime is that helicopter landing sites have to be larger and are often more distant from the incident location, requiring secondary transport by police from the landing site to the incident location.

### 2.4. Statistical Analysis

Data were analyzed with Stata 17.0 (StataCorp, College Station, TX, USA). The sample size of the BRAIN-PROTECT study was set at 2500 patients and is discussed in the study protocol [11]. The sample size for this secondary analysis was based on the available number of patients meeting the inclusion criteria in the study period. Descriptive statistics were used for demographic data, injury characteristics and outcome data (means, standard deviations, medians and quartiles or numbers and percentages as appropriate). Unadjusted differences between the day- and nighttime group were explored with the Mann–Whitney U test, *t*-test or chi-squared test. Exploratory unadjusted logistic regression analyses were performed on the association between day- versus nighttime treatments of TBI and 30-day mortality. After this, a multivariable logistic regression analysis adjusted for theoretical potential confounders was executed. Covariates included demographic factors (age, gender), the American Society of Anesthesiologists (ASA) score as an indicator of pre-injury healthcare status [12], markers of injury severity (prehospital Glasgow Coma Scale (GCS), Injury Severity Score (ISS)) and operational factors (distance from location to hospital, mechanism of injury, which of the four HEMS providers). Restricted cubic splines were modeled for the continuous variables age, GCS and ISS. To account for the non-independence of patients treated in the same hospital, cluster-robust standard errors were used in the regression models.

A planned subgroup analysis was performed for patients with confirmed TBI (head Abbreviated Injury Scale (AIS) ≥ 3) and isolated TBI (head ≥ 3, all other AIS < 3) regarding 30-day mortality.

A post hoc sensitivity analysis was executed, in which day- and nighttime were defined based on working hours instead on the UDP, to confirm the findings in the primary analysis. In this analysis, nighttime was defined as treatment during 6 p.m. until 6 a.m.

Secondary outcomes were analyzed using multivariable logistic regression with identical potential confounders in the model (intubation success rate), zero-truncated negative binomial regression (hospital LOS) and ordinal logistic regression (GOS at discharge). For all analyses, *p*-values < 0.05 were considered statistically significant.

## 3. Results

### 3.1. Baseline Characteristics

**Figure 1:**
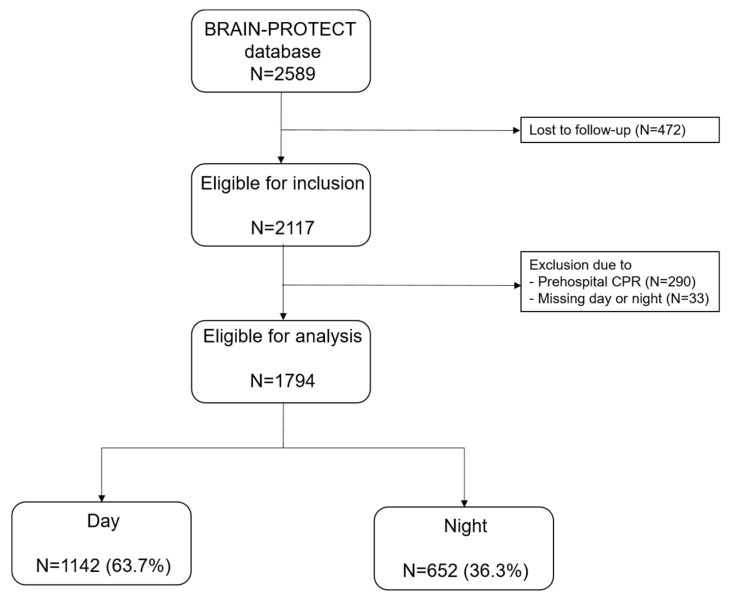
Flowchart of included patients.

The BRAIN-PROTECT database comprises 2589 patients with suspected severe TBI, of whom 1794 were eligible for analysis (Figure 1). Patients were excluded if no follow-up data were available (*n* = 472), if prehospital cardiopulmonary resuscitation was necessary (*n* = 290) or no data on day- or nighttime were available (*n* = 33). Table 1 shows the patient characteristics, mode and severity of injuries and outcome parameters stratified by treatment during day- or nighttime. The majority of the included patients were male (70.3%) with a median age of 45 (23–65) years, and 60.1% had a pre-injury ASA score of 1. Dispatches occurred more often during daytime (63.7%). The median GCS on arrival of HEMS was 4 (3–7).

### 3.2. Primary Outcome

Overall, the mortality rate after 30 days was 33.0%, with a lower percentage of death in the nighttime group than in the daytime group when compared in an unadjusted analysis (nighttime 28.7% versus daytime 35.5%, *p* = 0.004, Table 1). Univariable logistic regression analysis also showed a lower odds of 30-day mortality in the nighttime group (OR 0.74, 95% CI 0.60–0.91, *p* = 0.004). After adjusting for all potential confounders, the evidence for a difference in mortality between groups was no longer present (OR 0.82, 95% CI 0.59–1.16, *p* = 0.262) (Table 1 and Table 2). In the subgroup of patients with confirmed TBI, no difference was found in the mortality rate at 30 days (OR 0.89, 95% CI 0.60–1.31, *p* = 0.543). Patients with isolated TBI were less likely to die when treated by HEMS during nighttime than after accidents occurring during the day (OR 0.51, 95% CI 0.34–0.76, *p* = 0.001) after correcting for previously mentioned confounders. In the sensitivity analysis, the mortality of patients with isolated TBI was also lower during nighttime treatment, but this association failed to meet the significance criterion (OR 0.63, 95% CI 0.38–1.05, *p* = 0.077).

### 3.3. Secondary Outcomes

Functional outcome defined by the GOS at discharge was not associated with the time of treatment in the full cohort or in the subgroups of TBI (Table 3). Also, the length of hospital stay in patients surviving to discharge was not different between patients (incidence rate ratio (IRR) 0.90, 95% CI 0.80–1.01, *p* = 0.065). Prehospital endotracheal intubation was performed in the majority of patients (96.4% during daytime, 94.2% during night, *p* = 0.001).

## 4. Discussion

This retrospective analysis of the BRAIN-PROTECT database shows no difference in 30-day mortality after correction for potential confounders in the overall group of patients with suspected severe TBI when comparing prehospital HEMS treatment during day- or nighttime. Interestingly, in a subgroup analysis, patients with isolated severe TBI had lower mortality rates after treatment during nighttime than during daytime. Functional outcome was not associated with the time of presentation.

Our hypothesis was not supported. Specifically for patients with TBI, limited previous data are available. One observational study showed similar results and found no difference in hospital mortality and functional outcome at 6 months between patients presenting at office hours or any other time [8]. However, in another study, patients with severe TBI presenting at night had a longer time-to-surgery interval than during the day [13]. In the present analysis, patients with severe TBI presenting during daytime were significantly older than during nighttime. Previously, it has been shown that older age is associated with poorer outcome after TBI [14]. If the multivariable analysis was repeated without adjusting for age, nighttime treatment was associated with lower 30-day mortality (OR 0.65, CI 0.44–0.96, *p* = 0.031), emphasizing that patient-related factors play a major role in the overall outcome.

Most literature on the effect of after-hours, i.e., outside typical office hours, on outcome originates from studies in patients with cardiac arrest or general trauma. Specifically, for patients in cardiac arrest, in- or out-of-hospital, nighttime presentation is associated with worse outcome probably due to differences in availability of bystander CPR and ALS performance rates [15,16,17,18]. The data on the outcomes of general trauma patients admitted during the night are more conflicting. A database study in the United States in over 800,000 patients reported a higher in-hospital mortality in trauma patients arriving at night at a level-1 trauma center than during the day [19]. Also, two trauma registry studies from Asia showed higher mortality in the emergency department in patients presented at night [10,20]. In contrast, several observational trials reported no inferior outcomes among patients presenting during nighttime compared to those admitted during the day [21,22,23,24,25], even with high patient loads [26]. A number of studies investigating the national trauma system of a specific country also reported no differences in mortality or patient outcomes when comparing the time of day [27,28,29,30]. It must be noted that differences in the structure and system of prehospital care between countries may hamper the comparison of results. Also, many studies do not mention the duration of shifts of on-call personnel. The Dutch HEMS system from the present analysis had two shifts of 12.5 h per day. The availability and training level of HEMS staff is exactly the same during day and night. Also, it must be stressed that the present results not only reflect prehospital treatment but also in-hospital care. Level-1 trauma centers in the Netherlands are in general staffed and equipped to provide the highest level of care. However, some specialists may be on call from home during off hours. To better compare our results with previous studies, a sensitivity analysis was performed using actual working hours instead of the UDP, namely, from 6 a.m. to 6 p.m. In this analysis, in the overall patient group, 30-day mortality was similar between day- and nighttime treatments. So, nighttime treatment is not associated with worse patient outcome. This finding may only be extrapolated to countries with similar prehospital and in-hospital care to the Netherlands.

The second finding, that patients with isolated severe TBI had lower mortality rates at nighttime even after adjusting for theoretical confounders, was unexpected. In the Netherlands, a guideline is available for the prehospital treatment of patients with (isolated) severe TBI, and most of these patients are treated by one of four Dutch HEMS crews, regardless of the time of day or night. The patients were subsequently transported to nine different level-1 trauma centers. The travel time to the patient and travel time to a level-1 trauma center in the Netherlands were relatively short. This was an advantage as it has been shown that a longer prehospital time could be linked to worse functional outcome [7]. The Dutch infrastructure and the set-up of the trauma system in general could mean that the travel time to the hospital is shorter during the night in the relative absence of other traffic, resulting in earlier access to definitive surgical care. Our data indicate that the on-scene time was not different between day and night. Also, the time until the first CT scan was not different between the groups, indicating that the level of care in the first hours after the accident was similar between day and night. It could be argued that patients presenting at nighttime are more frequently under the influence of drugs and alcohol which might temporarily mimic TBI-related symptoms. No specific data on alcohol or drug use in this population are available. However, our subgroup analysis was based on the head AIS score, which was determined afterwards and implied actual severe traumatic brain injury. The head AIS does not take into account the specific type of brain injury and its location and severity. For example, acute subarachnoidal bleeding and the presence of a midline shift are indicators of worse patient outcome [14]. In the present analysis, differences in the type of brain injury between groups may have existed which may have influenced the results. The sensitivity analysis (applying a 6 a.m.–6 p.m. time frame) showed that the advantage of nighttime treatment in the subgroup of patients with isolated TBI was no longer statistically significant. This indicated that patient selection based on the UDP or actual time might, in part, explain the results.

Studying the effect of treatment during day- versus nighttime in a specific population is not a novel concept. However, this study is unique in its design to also include prehospital treatment and not just in-hospital care. For many patient groups and diseases, just as for patients with severe TBI, treatment starts before the patient reaches the hospital. By combining prehospital and in-hospital care during both day and night, more relevant factors affecting patient outcome could be studied.

The present study is a retrospective analysis of prospectively collected data. Common limitations involved are selection and information bias. Although efforts were made in the design of the original study to minimize such bias as described previously in detail [11], it cannot be ruled out that the results were affected. Inherent to observational studies, findings must be interpreted as associations and not as causal relationships. The patients were divided into day- and nighttime based on the time of dispatch in relation to the UDP. This is somewhat different from other studies were subgroups were formed based on weekdays versus weekends, before or after midnight, etc. The focus of our research was on prehospital HEMS treatment, acknowledging that after the UDP, regulations for HEMS operations are more strict which may have an influence on the travel time to the patient and other aspects relevant to patient outcome. Since the UDP is not a fixed time period in both the start/end and in duration, it may have further influenced the results. For example, in the winter, darkness covers the rush hours in the morning and evening, whereas in summer, rush hours are in daytime.

## 5. Conclusions

In conclusion, the prehospital nighttime treatment of patients with severe TBI combined with any other injury was not associated with worse outcome. Patients with isolated severe TBI had lower mortality rates at 30 days when treated at nighttime versus daytime. Future studies focusing on the operational and medical–technical aspects of prehospital treatment could shed a further light on this association.

## Figures and Tables

**Table 1:** Patient characteristics.

	Overall *n* = 1794	Day *n* = 1142	Night *n* = 652	*p*-Value	Missing
*Demographic data*					
Age (years) ^s^	45 (23–65)	50 (24–68)	34 (22–57)	<0.001	20
Male sex (*n* (%))	1259 (70.3)	792 (69.4)	457 (71.8)	0.279	3
ASA pre-injury ^s^				0.007 ^	347
ASA 1	874 (60.1)	528 (57.3)	346 (65.0)		
ASA 2	398 (27.4)	273 (29.6)	125 (23.5)		
ASA 3	182 (12.5)	121 (13.1)	61 (11.5)		
Mechanism of injury ^s^				0.005 ^	29
MVA	310 (17.2)	154 (13.4)	156 (23.9)		
Motorcycle	161 (8.9)	102 (8.9)	59 (9.0)		
Bicycle	393 (21.9)	291 (25.4)	102 (15.6)		
Pedestrian	122 (6.8)	72 (6.3)	50 (7.6)		
Traffic other	54 (3.0)	34 (2.9)	20 (3.0)		
Fall from height	622 (34.6)	396 (34.6)	226 (34.6)		
Gunshot/stab injury	34 (1.8)	22 (1.9)	12 (1.8)		
Other	69 (3.8)	50 (4.3)	19 (2.9)		
GCS at arrival ^s^	4 (3–7)	5 (3–7)	4 (3–7)	0.212 ^	0
ISS ^s^	26 (18–34)	26 (20–35)	26 (17–34)	0.243	208
*Pre-/in-hospital data*					
Distance (km)	24.2 (13.7–40.1)	25.4 (15.7–41.9)	22.1 (9.9–37.1)	<0.001	293
Prehospital endotracheal intubation				0.001	
No	56 (3.1)	23 (2.0)	32 (4.9)		1
Yes successful	1709 (93.5)	1071 (93.8)	607 (93.1)		31
Yes unsuccessful	38 (2.1)	30 (2.6)	7 (1.1)		1
On-scene time (min)	16 (11–24)	16 (11–25)	16 (11–24)	0.426 #	557
Time to first CT scan	22 (16–30)	22 (16–31)	22 (16–30)	0.377	444
*Outcome*					
30-day mortality ^±^	555 (33.0)	378 (35.5)	177 (28.7)	0.004	111
Hospital LOS (days) *	9 (2–23)	8 (2–23)	10 (2–24)	0.502 ^	137
GOS at discharge				0.015	
Death	571 (31.2%)	382 (33.5%)	178 (27.9%)		11
Neurovegetative	38 (2.1%)	25 (2.2%)	13 (2.0%)		0
Severe disability	596 (32.9%)	372 (32.6%)	212 (33.2%)		12
Moderate disability	175 (9.7%)	105 (9.2%)	68 (10.6%)		2
Good recovery	266 (14.7%)	147 (12.9%)	115 (18.0%)		4
TBI category					
Confirmed	1364	869 (86.6%)	468 (82.25%)	0.019	
Isolated	713	455 (45.4%)	248 (43.5%)	0.477	

Numeric variables are presented as mean (SD) or median (quartiles). Percentages are calculated per column; rounding error and missing data may account for not adding up to 100%. ASA: American Society of Anesthesiologists, GCS: Glasgow Coma Scale, GOS: Glasgow Outcome Scale, ISS: Injury Severity Score, MVA: motor vehicle accident. * Hospital LOS is analyzed only in patients known to survive until discharge. ^±^ Univariable logistic regression analysis of day/nighttime vs. 30-day mortality. ^ Ranksum test. * Chi 2. # *t*-test with unequal variances assumed. ^s^ Univariable logistic regression with *p* < 0.05 for association with 30-day mortality.

**Table 2:** Daytime versus nighttime treatment and 30-day mortality.

Logistic Regression	OR	95% CI	*p*-Value
*Primary analysis—UDP—univariable*			
All patients	0.74	0.60–0.91	0.004 *
*Primary analysis—UDP—multivariable*			
All patients	0.82	0.59–1.16	0.262
Confirmed TBI	0.89	0.60–1.31	0.543
Isolated TBI	0.51	0.34–0.76	0.001 *
*Sensitivity analysis—6 p.m. to 6 a.m.—multivariable*			
All patients	0.82	0.56–1.20	0.316
Confirmed TBI	0.88	0.57–1.35	0.566
Isolated TBI	0.63	0.38–1.05	0.077

Multivariable logistic regression analysis of the association between daytime versus nighttime treatment and mortality within 30 days. CI: confidence interval, OR: odds ratio, UDP: universal daylight period, TBI: traumatic brain injury. * Below significance threshold of 0.05 (two-sided).

**Table 3:** Secondary outcomes.

Logistic Regression	OR	95% CI	*p*-Value
*Glasgow Outcome Scale*			
All patients	1.16	0.89–1.52	0.262
Confirmed TBI	1.02	0.72–1.43	0.921
Isolated TBI	1.36	0.88–2.09	0.168
*Hospital length of stay*	**IRR**		
All patients	0.90	0.80–1.01	0.065
Confirmed TBI	0.92	0.83–1.02	0.097
Isolated TBI	0.87	0.76–0.99	0.033 *

Multivariable logistic regression analysis of the association between daytime versus nighttime treatment and Glasgow Outcome Scale (GOS) at discharge and hospital length of stay. Variables in model: age (spline), gender, Glasgow Coma Scale (spline), ISS (spline), injury mechanism, distance between incident and trauma center (spline) and P-HEMS station. CI: confidence interval, IRR: incidence rate ratio, OR: odds ratio, TBI: traumatic brain injury. * Below significance threshold of 0.05 (two-sided).

## Data Availability

Data are contained within the article.
